# Author Correction: Longitudinal urinary biomarkers of immunological activation in covid-19 patients without clinically apparent kidney disease versus acute and chronic failure

**DOI:** 10.1038/s41598-021-01792-4

**Published:** 2021-11-11

**Authors:** Krzysztof Laudanski, Tony Okeke, Jihane Hajj, Kumal Siddiq, Daniel J. Rader, Junnan Wu, Katalin Susztak

**Affiliations:** 1 Department of Anesthesiology and Critical Care, The University of Pennsylvania, Philadelphia, PA USA; 2 Leonard Davis Institute for Healthcare Economics, The University of Pennsylvania, Philadelphia, PA USA; 3 School of Biomedical Engineering, Science and Health Systems, Drexel University, Philadelphia, PA USA; 4 School of Nursing, Widener University, Philadelphia, PA USA; 5 College of Arts and Sciences, Drexel University, Philadelphia, PA USA; 6 Department of Genetics, Perelman School of Medicine, The University of Pennsylvania, Philadelphia, PA USA; 7 Division of Renal Electrolyte and Hypertension, Department of Medicine, The University of Pennsylvania, Philadelphia, PA USA

Correction to: *Scientific Reports* https://doi.org/10.1038/s41598-021-99102-5, published online 04 October 2021.

The original version of this Article contained an error in the spelling of the author Jihane Hajj which was incorrectly given as Hajj Jihane.

The original Article has been corrected.

